# Sinoatrial node dysfunction induces cardiac arrhythmias in diabetic mice

**DOI:** 10.1186/s12933-014-0122-y

**Published:** 2014-08-12

**Authors:** Ewa Soltysinska, Tobias Speerschneider, Sine V Winther, Morten B Thomsen

**Affiliations:** From the Danish National Research Foundation Centre for Cardiac Arrhythmia, Department of Biomedical Sciences, Faculty of Health and Medical Sciences, University of Copenhagen, Blegdamsvej 3b; bldg.: 12.5.36, Copenhagen, DK-2200 Denmark

**Keywords:** Sinus node, Heart rate variability, ECG, Sympathetic nervous system

## Abstract

**Background:**

The aim of this study was to probe cardiac complications, including heart-rate control, in a mouse model of type-2 diabetes. Heart-rate development in diabetic patients is not straight forward: In general, patients with diabetes have faster heart rates compared to non-diabetic individuals, yet diabetic patients are frequently found among patients treated for slow heart rates. Hence, we hypothesized that sinoatrial node (SAN) dysfunction could contribute to our understanding of the mechanism behind this conundrum and the consequences thereof.

**Methods:**

Cardiac hemodynamic and electrophysiological characteristics were investigated in diabetic db/db and control db/+ mice.

**Results:**

We found improved contractile function and impaired filling dynamics of the heart in db/db mice, relative to db/+ controls. Electrophysiologically, we observed comparable heart rates in the two mouse groups, but SAN recovery time was prolonged in diabetic mice. Adrenoreceptor stimulation increased heart rate in all mice and elicited cardiac arrhythmias in db/db mice only. The arrhythmias emanated from the SAN and were characterized by large RR fluctuations. Moreover, nerve density was reduced in the SAN region.

**Conclusions:**

Enhanced systolic function and reduced diastolic function indicates early ventricular remodeling in obese and diabetic mice. They have SAN dysfunction, and adrenoreceptor stimulation triggers cardiac arrhythmia originating in the SAN. Thus, dysfunction of the intrinsic cardiac pacemaker and remodeling of the autonomic nervous system may conspire to increase cardiac mortality in diabetic patients.

## Background

Diabetes mellitus is a serious public health concern and the global prevalence was estimated to be 2.8% in 2000 rising to 4.4% in 2030 [1]. The incidence of cardiovascular disease is higher among diabetic patients [2], and they often die from cardiovascular complications [3]. Coronary artery disease secondary to atherosclerosis account for a large fraction of the comorbidity, likely underlying the reports of myocardial infarction as the main cause of mortality in diabetic patients [3]. The vast majority of diabetic patients have type-2 diabetes mellitus (T2DM), characterized by obesity, insulin resistance and very high blood glucose levels.

Elevated resting heart rate is associated with an increased risk of cardiovascular complications and sudden cardiac death in the general population and in T2DM patients [4–6]. Conversely, in patient groups with electronic cardiac pacemakers due to slow heart rates, there is a statistically significant overrepresentation of diabetic patients, suggesting diabetes-induced impairment of the endogenous, natural pacemaker of the heart [7–9]. Sick sinus node syndrome is associated with T2DM in case reports only [10]. Reports of impaired atrio-ventricular node in diabetic patients [11,12] supports the clinical observation that the cardiac conduction system, especially the function of the nodes, are compromised in T2DM. Hence, there are strong clinical indications of altered sino-atrial node (SAN) function in T2DM and that this could contribute to the increased cardiovascular mortality in this large patient population.

Leptin-receptor deficient homozygous db/db mouse lacks the hypothalamic leptin regulation resulting in development of obesity and severe T2DM [13]. The model shows age-dependent progression of metabolic abnormalities that mimics the pathogenesis of T2DM in humans, including early insulin resistance and hyperinsulinemia at mouse age 6–12 weeks, followed by an insulin-secretory defect and hypoinsulinemia after 12 weeks [14]. Previous experimental studies related to effects of diabetes on SAN function have been conducted primarily in T1DM animal models [15–17], which lack the metabolic complexity of T2DM. Reports from heart rate recordings in conscious or anesthetized db/db mice with T2DM have revealed profound inconsistencies in results with examples of faster [18], slower [19,20] and comparable [21–23] resting heart rates. The clinical finding of SAN dysfunction in a small subset of diabetic patients and the discrepancies in the experimental findings of heart rate in db/db mice imply that this mouse model may constitute a unique tool for studying the effects of diabetes on SAN function.

In the present study, we hypothesized that db/db mice have a concealed SAN dysfunction in vivo that can be unmasked by the appropriate stress. We probed and challenged SAN function to elicit changes in heart rate and heart rate instability compatible with the clinical observations of augmented cardiovascular death. At basal conditions, heart rate is not different in anesthetized db/db mice; however, upon a challenge with β-adrenoreceptor stimulation we exposed pronounced SAN dysfunction inducing prominent heart-rate fluctuations. Invasive electrophysiological studies showed a prolonged sinus node recovery time and immunoblots of SAN tissue identified reduced density of autonomic nerve endings.

## Methods

### Animals

Experiments were performed using male leptin-receptor deficient db/db mice (C57BL/KS-lepr^*db*^/lepr^*db*)^ and lean control heterozygote db/+ mice (C57BL/KS-lepr^*db*^/lepr^+^), aged 14–16 weeks. Animals were purchased from Taconic (Denmark) and housed in a specific pathogen free facility with *ad libitum* access to water and standard chow food in a room with a 12-h light/dark schedule and an ambient temperature of 22°C. Mice were anesthetized in 1.5-2% isoflurane in 100% O_2_. Body temperature was constantly monitored and kept at 37 ± 0.5°C. Blood glucose levels were determined from the tail vein as the mean of 2 consecutive measurements by a Microdot glucometer (Kacey Diagnostics, USA). Body weight was determined prior to each investigation. At the end of experiments, euthanasia was achieved by cervical dislocation. A total of 12 db/db mice and 12 db/+ mice were used for this study. The study conformed to the *Principles of laboratory animal care* (National Institutes of Health, revised 1996) and was approved by the national ethics committee.

### Echocardiography

Transthoracic echocardiography was performed with a linear 15–45 MHz transducer (Vevo 770, VisualSonics, Canada), as previously described [24]. Inner diameter of the left ventricle (LVID) and LV wall thicknesses (LVWT) in diastole and systole were measured in M mode at the maximal and minimal cross sectional diameter in a heart cycle, respectively, at the level of the Papillary muscles and averaged from parasternal long axis and short axis view. Left ventricular length from aortic valve to LV endocardial apex was measured in the parasternal long axis view. Left ventricular end-diastolic volume (LVEDV) and end-systolic volume (LVESV) were calculated as LVID^3^*{7/(2.4 + LVID)} in diastole and systole, respectively. Stroke volume (SV = LVEDV-LVESV) and ejection fraction (EF = SV/LVEDV) were calculated. Subsequently, LV filling properties were evaluated by pulsed wave Doppler imaging. Recordings were made at the tips of the mitral-valve leaflets in the apical four-chamber view. Peak early (E) and late (A) mitral inflow velocities, deceleration time (DT) of early filling and isovolumetric relaxation time (IVRT) and contraction time (IVCT) were measured as described previously [25]. Three measurements from consecutive heart cycles were obtained and averaged in each mouse. Six mice of each genotype were used in the echocardiographic study.

### Electrocardiography

A 6-lead surface electrocardiogram (ECG) was recorded (LabChart 8, ADInstruments, Australia) using subcutaneous needle electrodes. Signals were recorded at 4 kHz and filtered using a low-pass setting of 1 kHz and a high-pass setting of 0.3 Hz. After 10 min baseline, isoprenaline (2 μg/g) was injected intraperitoneally. Averaged signal ECGs were generated by aligning R waves of >200 complexes from lead I and analyzed manually as previously described in detail [26]. The dominant cardiac depolarization axis was determined from vectorcardiography from the frontal plane as the angle between lead I and the largest amplitude vector during the QRS complex. Mice were challenged with a β-adrenoreceptor agonist, isoprenaline, administered intraperitoneally at a dose of 2 μg/g body weight as previously described [24]. Isoprenaline (Sigma-Aldrich, St. Louis, MO) was dissolved and diluted in saline on the day of experiment.

In order to quantify fluctuation in RR intervals and SAN arrhythmias, we used the algorithms of heart-rate variability (HRV). Consecutive RR intervals were determined in comparable time epochs of 10 minutes at baseline and at 5–10 minutes after β-adrenoreceptor stimulation, and the following parameters were calculated [27]: standard deviation of RR intervals (SDRR), standard deviation of the difference between successive RR intervals (SD∆RR), percentage of normal consecutive RR intervals differing by >6 ms (pRR6, see reference [27]), normalized power contained within the low frequency range (0.15-1.5 Hz, LF power), and normalized power contained within the high frequency range (1.5-5.0 Hz, HF power). The presence of induced SAN arrhythmia in a mouse was acknowledged if SDRR, SD∆RR or pRR6 increased by >100% following β-adrenoreceptor stimulation.

Twelve mice of each genotype were used in the electrocardiographic study, half of which had been used prior in the echocardiographic study. Three db/db mice and one db/+ mouse died within a 12-hour period after recovery from the electrocardiographic study; the remaining 9 db/db and 11 db/+ mice were used subsequently for an invasive electrophysiological study.

### Intracardiac pacing

Mice were fasted for 16 hours prior to intracardiac pacing to challenge the mice with hypoglycemia. During anesthesia, a 1.1 F pacing catheter (EPR-800, ADInstruments) was advanced into the LV through a small incision in the right common carotid artery. Atria and ventricles were paced at 1.5 and 2 times capture threshold amplitudes, respectively. Sinus node recovery time (SNRT) was measured twice after delivering an atrial pacing train with a cycle length of 100 ms (600 beats per minute) for 15 seconds, and defined as the duration between the last pacing stimuli and the onset of the first spontaneous P wave. Rate-corrected SNRT (CSNRT) was calculated by subtracting the baseline, sinus-node controlled PP interval length from the SNRT. Wenckebach periodicity was determined by progressively shortening the paced cycle length and defined as the shortest cycle length maintaining a 1:1 conduction between the atrium and ventricles. Ventricular effective refractory period (VERP) was determined by applying 9 paced ventricular beats (9xS1) at a cycle length of 100 ms followed by a single extra-stimulus (S2) that was sequentially increased in 1 ms steps until capture. VERP was defined as the longest S1-S2 interval that did not result in ventricular capture. Inducibility of arrhythmia was tested by a pacing protocol consisting of 9xS1 at the cycle length of 100 ms followed by 6xS2 [24]. Ventricular tachycardia (VT) was defined as ≥4 spontaneous consecutive beats following the last captured S2. If VT was induced, the stimulation protocol was repeated twice to ascertain reproducibility [24].

### Immunoblotting

The right atria were homogenized in ice-cold RIPA buffer supplemented with protease inhibitors (AEBSF, 2 mM; aprotinin, 0.3 μM; bestatin, 130 μM; E-64, 14 μM; leupeptin, 1 μM) using the Precellys system (Bertin Technologies, France). Proteins (10 μg) were separated on a 4-15% SDS-PAGE (Biorad, Denmark) and blotted onto hybond-P polyvinylidene fluoride transfer membranes (Amersham Biosciences, Denmark). Membranes were incubated with anti-synaptophysin antibody (0.2 μg/ml, Santa Cruz Biotechnology, USA). Synaptophysin is essential for synaptic vesicle regulation neurons and is a marker of nerve synapses. Immunoreactive proteins were detected by HRP-linked donkey anti-rabbit antibody (8 ng/ml, Jackson Immunosearch Laboratories, UK). Membranes were stripped and re-probed with anti-actin antibody (MAB1501, Millipore, Denmark). Protein band density was quantified as a Gaussian densiometric trace on the exposed films and processed equally. Immunoblotting was repeated three times and the average ratio of Synaptophysin/Actin bands density was calculated for each sample and used for the comparison between groups.

### Statistical analysis

Data is represented as mean ± SEM and the number of observations (n) is indicated on each figure. Two-tailed Student’s *t*-test and two-way ANOVA followed by post-hoc Bonferroni *t*-test were used for comparison of 2 or more groups, respectively. Fisher’s exact test was employed to compare the occurrence of arrhythmias. P-values <0.05 were considered statistically significant.

## Results

### Improved systolic function and reduced diastolic function in diabetic mice

**Figure 1:**
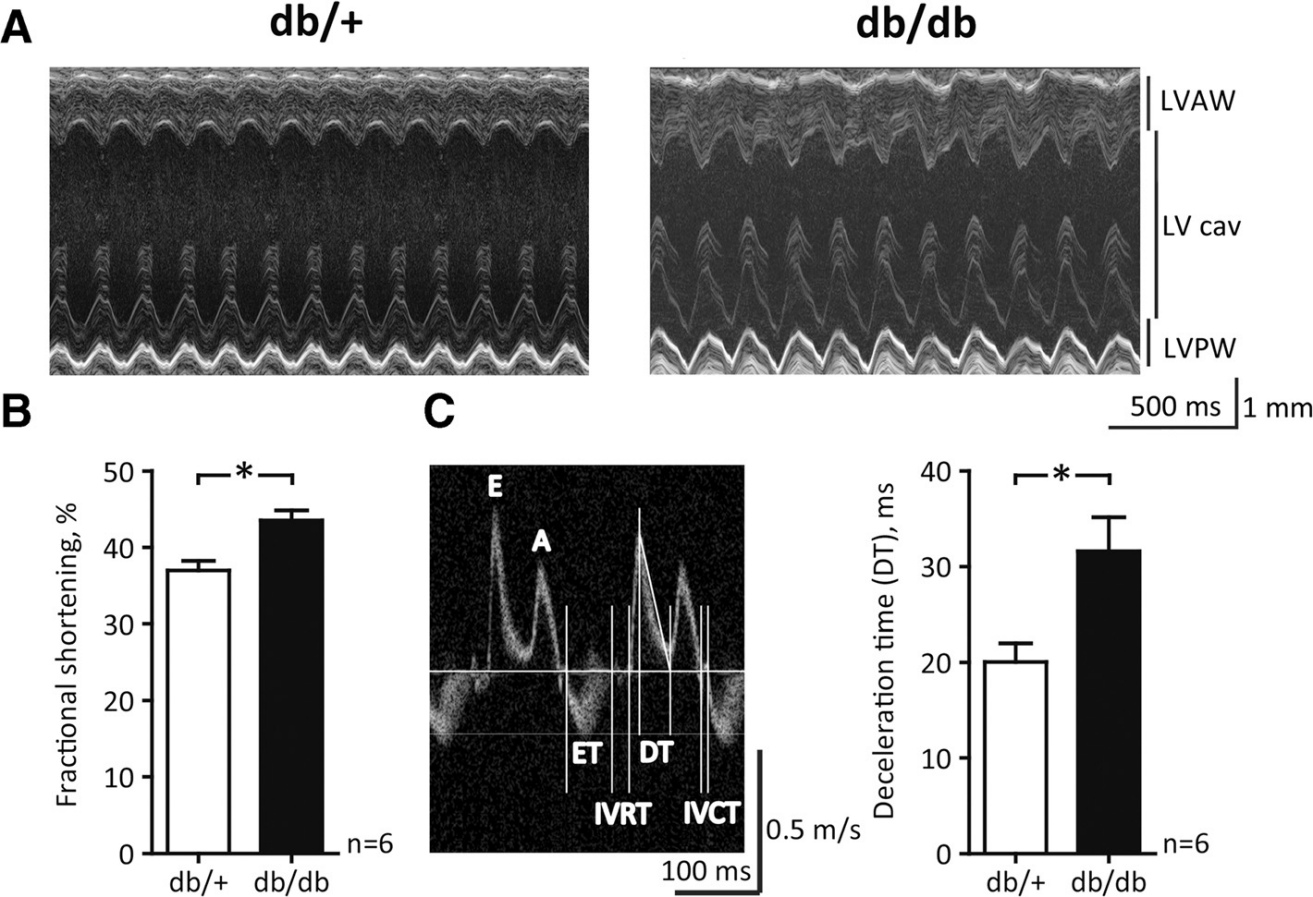
**Echocardiographic assessment of left ventricular structure and mechanical function. A**, Representative M-mode echocardiographic images of the left ventricle (LV) from anesthetized mice. LV cav: cavity; LVAW: anterior wall; LVPW: posterior wall. **B**, Fractional shortening of the LV. **C**, Left panel: Exemplary transmitral velocity-time curves with positive peaks representing flow velocities during passive early filling (E) and atrial contraction (A). Vertical lines indicate the demarcation of ejection time (ET), deceleration time (DT), isovolumetric relaxation time (IVRT) and isovolumetric contraction time (IVCT). Right panel: Deceleration phase of the E peak. *, P < 0.05, Student’s *t*-test.

At the age of 14–16 weeks, db/db mice exhibited pronounced hyperglycemia and larger body weights (Table 1). Wet cardiac weights were lower in db/db mice (Table 1). To evaluate LV structure and mechanical function cardiac imaging was performed in the intact animal (Figure 1A). We noted no difference in the LV wall thickness (db/+: 0.92 ± 0.06 versus db/db: 0.86 ± 0.03 mm in diastole; n = 6, P > 0.05); however, db/db mice have significantly shorter LV length than db/+ mice (db/+: 7.3 ± 0.1 versus db/db: 6.8 ± 0.2 mm in diastole; n = 6, P < 0.05).

**Table 1:** **Characteristics of the control (db/+) and diabetic (db/db) mice**

	**db/+ (n = 11)**	**db/db (n = 9)**
Body weight, g	26 ± 0.5	39 ± 1.5*
Heart weight, mg	171 ± 5.5	145 ± 4.5*
Tibia length, mm	21 ± 0.3	20 ± 0.4
Non-fasting blood glucose, mg/dL	190 ± 13	526 ± 26*
Fasting blood glucose, mg/dL	76 ± 98	474 ± 42*

*, P < 0.05, Student’s *t*-test.

A hypercontractile systolic function in db/db mice was indicated by a greater fractional shortening (Figure 1B) and larger ejection fraction (db/+: 67 ± 2 versus db/db: 75 ± 1%; n = 6, P < 0.05). This is further supported by Doppler analysis of mitral inflow, where isovolumetric contraction time (IVCT) tended to be shorter in db/db mice (db/+: 11.4 ± 0.3 versus db/db: 10.7 ± 0.2 ms; n = 6, P = 0.07), suggesting improved systolic function in db/db mice. In contrast, isovolumetric relaxation time (IVRT) was indistinguishable (db/+: 22.2 ± 3.5 versus db/db: 20.8 ± 2.7 ms; n = 6, P > 0.05). Similarly, analysis of Doppler-derived peak velocities during early (E) ventricular filling and atrial (A) contraction (Figure 1C, left panel) showed no difference between genotypes (E/A ratio in db/+: 1.59 ± 0.04 versus db/db 1.88 ± 0.02; n = 6, P > 0.05); however, deceleration time of early peak flow (DT) was prolonged by 55% in db/db mice suggesting a diastolic insufficiency in these mice (Figure 1C). Taken together, a shorter heart, improved systolic function and reduced diastolic function indicate early ventricular remodeling in db/db mice.

### Unexpected electrophysiological phenotype in db/+ mice

**Figure 2:**
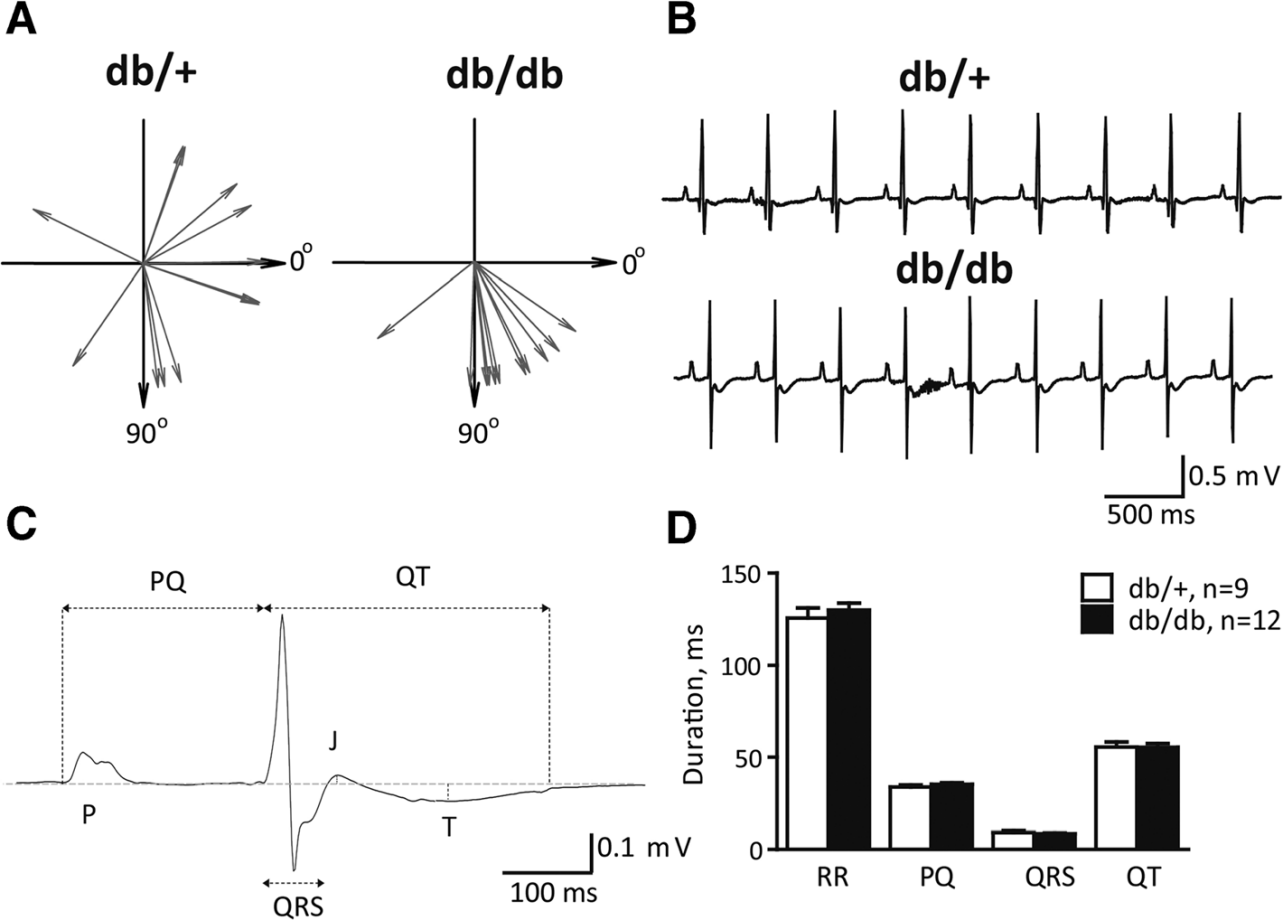
**Electrophysiological function. A**, Cardiac axis in individual mice. **B**, Raw ECG traces from lead I in db/+ and db/db mice. **C**, Exemplary averaged ECG complex in a db/db mouse shown on the enlarged scale. **D**, No statistical difference of averaged ECG intervals was found between db/+ and db/db mice.

Next, we evaluated electrophysiological function by analyzing surface ECG in anesthetized animals (Figure 2). The cardiac QRS axis was normal and uniform in most db/db mice (75 ± 8°; n = 12); however in db/+ mice we observed dominant axes in all 4 quadrants of the vectorcardiogram (Figure 2A). On average, the cardiac axis in db/+ mice was 3 ± 23° (n = 12; P < 0.05 versus db/db), where the large standard error reflects the very heterogeneous group of mice. Indeed, we excluded 3 db/+ mice from further electrocardiographic comparison due to incomparable ECGs (cardiac axes in these mice were −154°, −28° and +125°, respectively). A representative raw ECG trace in a db/db mouse and an example with comparable ECG morphology from a db/+ mouse, are depicted on Figure 2B.

Analysis of averaged ECG signals (Figure 2C), showed no difference in the duration of RR, PQ, QRS and QT intervals (Figure 2D). Heart rates were 485 ± 20 and 465 ± 13 beats per minute in db/+ and db/db mice, respectively (P > 0.05). The amplitude of the J wave was comparable in two groups of mice (db/+: −51 ± 8 versus db/db: −47 ± 9 μV; P > 0.05). The amplitude of T wave could be reliably measured in 11/12 db/db mice but only in 4/9 control mice due to highly variable cardiac axes and consequently heterogeneous ECG morphologies. Based on this limited number of observations, the peak of T wave was more negative in the db/db versus db/+ mice (db/+: −30 ± 3 versus db/db: −55 ± 6 μV; P < 0.05).

### Compromised sinoatrial function in diabetic mice

**Figure 3:**
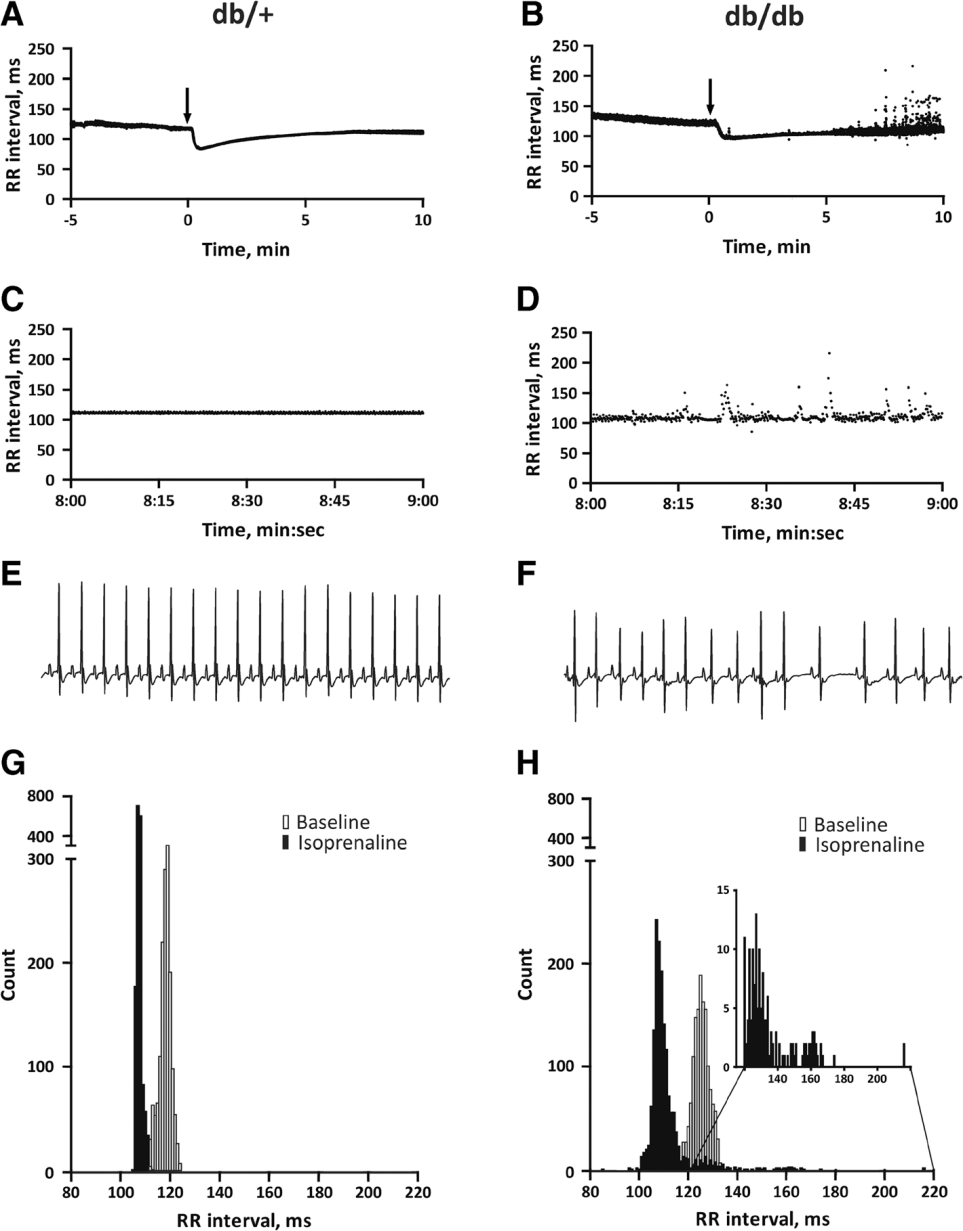
**Dynamics of RR interval upon β-adreneroceptor stimulation. A**, **B** Exemplary tachograms of RR intervals at baseline and during isoprenaline challenge (arrows indicate IP administration of isoprenaline). **C**, **D** The same two tachograms at enlarged scales, showing RR intervals during 1 minute (8 to 9 minutes after administration of isoprenaline). **E**, **F** Two-second ECG traces from the same mice, around time point 8:40 after administration of isoprenaline. **G**, **H** Histograms of RR intervals constructed by analysis of 3 minutes ECG recordings before and after isoprenaline administration.

Within the individual mouse, the ECG complexes were comparable and very regular at baseline in both groups of mice (Figure 2B), revealing no indications of SAN dysfunction. β-Adrenoreceptor stimulation significantly increased heart rate in both db/+ and db/db mice in a comparable manner (Figure 3A and B; mean heart rate increase were db/+: 28 ± 5 versus db/db: 32 ± 4% increase; P > 0.05). The inter-beat intervals remained relatively constant after isoprenaline administration in the control mouse (Figure 3A and C); however, the RR tachogram in the db/db mouse (Figure 3B and D) showed a large scatter of inter-beat intervals 5–10 minutes after β-adrenoreceptor challenge. The ECG in the Figure 3F clearly shows that the arrhythmia originated from the SAN as the observed RR fluctuations were accompanied by preserved P waves and PQ intervals. The histograms shown in Figure 3G and H show that the distributions of inter-beat intervals were comparable in two representative mice at baseline. After challenge with isoprenaline, the range of RR intervals in the control mouse remained narrow (range: 105–113 ms); however, in contrast, db/db mice show a wide spread of RR intervals (range: 85 to 216 ms). In particular, we recorded multiple late beats, in the range of 120–220 ms, (inset on the Figure 3H) which were never seen in any control mice.

**Figure 4:**
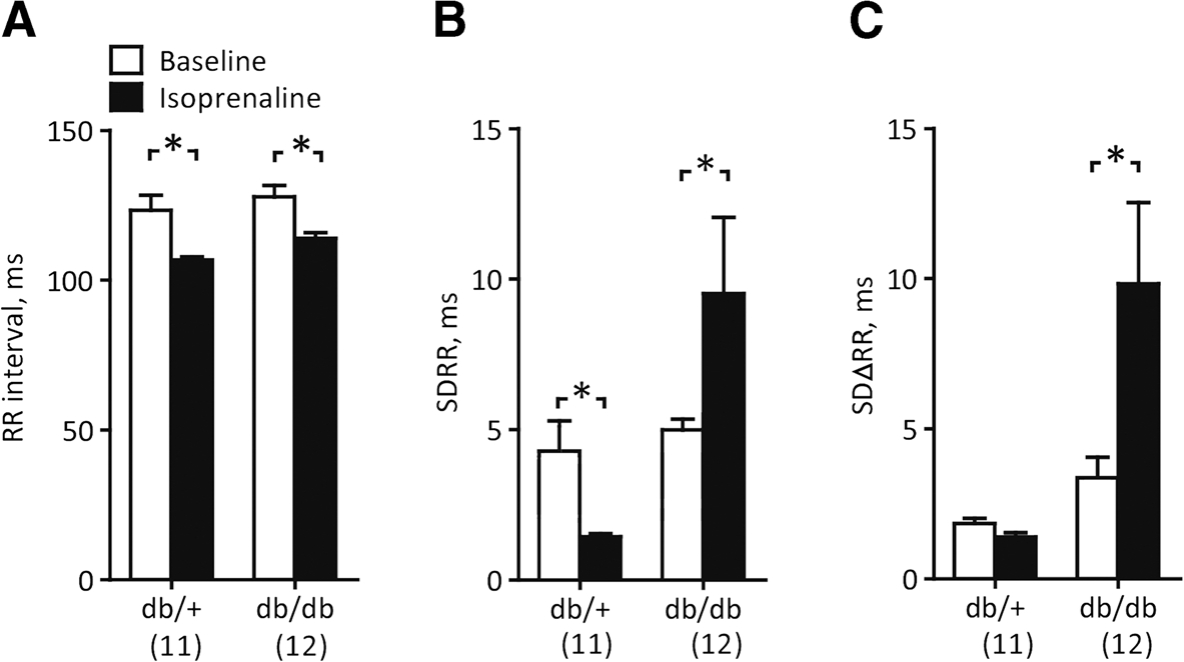
**Quantification of RR fluctuations. A** Mean RR interval during 3-min periods before and after isoprenaline injection. **B** Standard deviation of the same RR intervals. **C** Standard deviation of the differences between consecutive RR intervals. *, P < 0.05, Repeated-measures two-way ANOVA followed by post-hoc Bonferroni test.

To quantify these SAN arrhythmias, we took advantage of standard HRV algorithms. Before β-adrenoreceptor stimulation, RR, SDRR and SDΔRR were indistinguishable in db/+ and db/db mice (Figure 4, Table 2). After β-adrenoreceptor stimulation, these HRV parameters remained unchanged in control mice, but increased dramatically in db/db mice (Figure 4B and C). Altogether, episodes of SAN arrhythmia were observed in 7/12 db/db mice but in none of the control animals (P < 0.05). During recovery from the electrophysiological study, 3 db/db mice and 1 db/+ mouse died. We have no ECG record of the events.

**Table 2:** **Heart-rate variability parameters obtained in anesthetized control (db/+) and diabetic (db/db) mice before and after 16 hours fasting**

	**Db/+, fed**	**Db/db, fed**	**Db/+, fasted**	**Db/db, fasted**
RR, ms	124 ± 4	131 ± 3	137 ± 4 †	154 ± 4*†
HR, bpm	483 ± 17	458 ± 12	438 ± 11 †	390 ± 10*†
SDRR, ms	4.4 ± 1.0	5.0 ± 0.9	5.3 ± 0.8	6.0 ± 1.6
SD∆RR, ms	2.1 ± 0.3	3.2 ± 0.6	2.8 ± 0.6	4.7 ± 0.7*
pRR6,%	3 ± 4	11 ± 5^a^	8 ± 3	22 ± 6*
LF Power, μs^2^	0.9 ± 0.2	0.9 ± 0.4	1.0 ± 0.4	2.5 ± 1.0
HF Power, μs^2^	2.4 ± 0.6	7.9 ± 3.8	6.1 ± 3.4	15.4 ± 4.3^a^
LF Power, %	32 ± 4	17 ± 4	17 ± 4	15 ± 5
HF Power, %	67 ± 4	84 ± 4	84 ± 4	86 ± 5
LF/HF	55 ± 11	25 ± 10	24 ± 9	19 ± 4
n	12	12	10	9

*Abbreviations: HR (bpm)* heart rate in beats per minute, *SDRR* standard deviation of RR intervals, *SD∆RR* standard deviation of the difference between successive RR intervals, *pRR6* percentage of normal consecutive RR intervals differing by >6 ms, *LF power* absolute and normalized power contained within the low frequency range (0.15-1.5 Hz), *HF power* absolute and normalized power contained within the high frequency range (1.5-5.0 Hz). See reference [27]. Two-way ANOVA with a post hoc Bonferroni *t* test when appropriate. *, P < 0.05 versus db/+ mice; †, P < 0.05 versus fed mice of the same genotype; ^a^, P = 0.063 versus db/+. One mouse was excluded from the db/+, fasted group due to technical difficulties with anesthesia.

In order to challenge the mice with relative hypoglycemia, we subjected the mice to 16-hours fasting which decreased blood glucose concentration in both db/+ and db/db (Table 1). Reduction of glucose concentration had no effect on ECG morphology, but we noted a modest but significant increase of RR intervals as a consequence of fasting (Table 2). Hypoglycemia resulted in dramatic increases of SDΔRR and pRR6 in db/db mice only (Table 2). Spectral analysis of HRV showed no difference in autonomic control of heart rate at baseline during anesthesia (Table 2).

**Figure 5:**
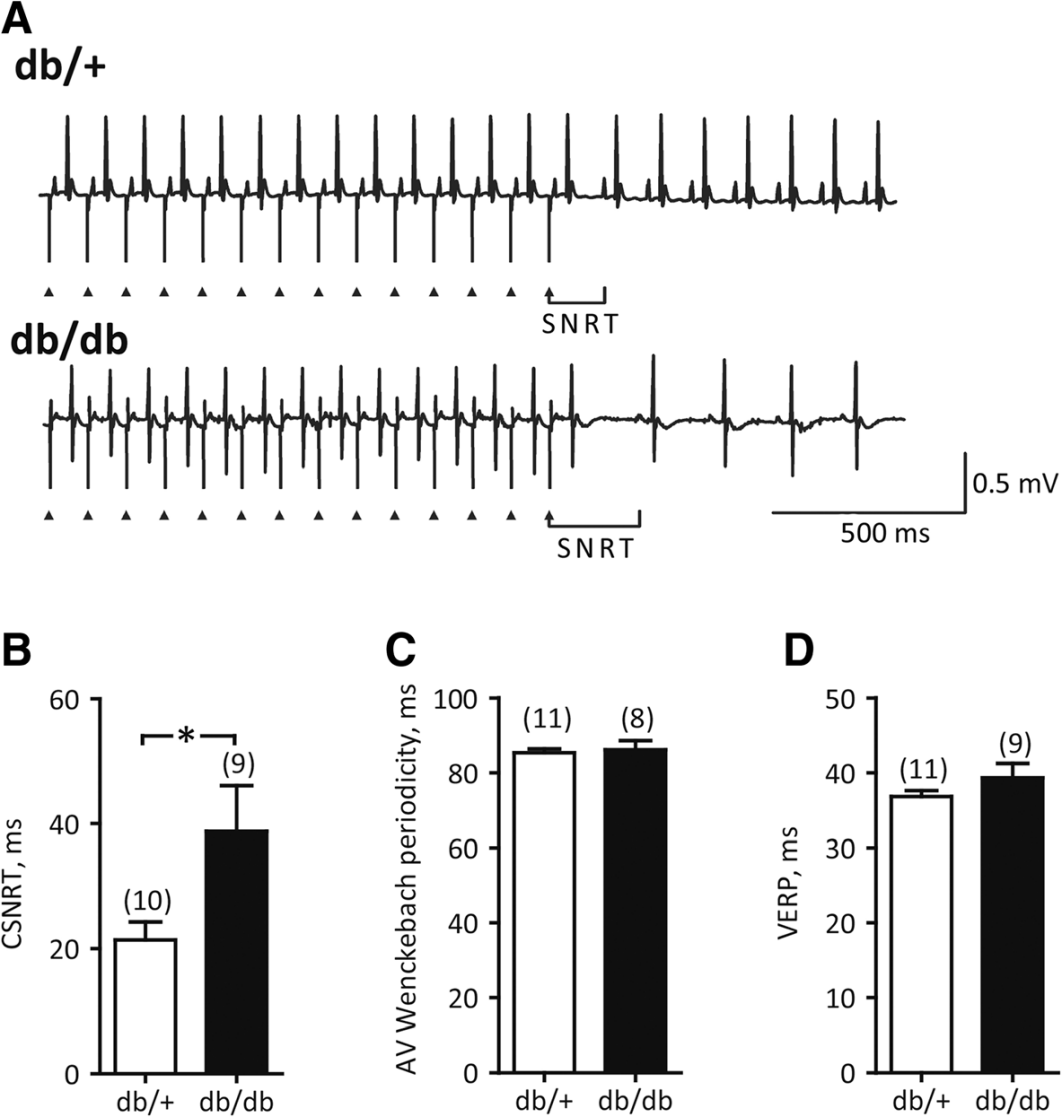
**Invasive electrophysiological study. A**, Measurements of sinus node recovery time (SNRT): exemplary ECG recordings obtained during atrial pacing at 100 ms paced cycle length and immediately upon termination of electrical stimulation. Triangles indicate stimulus artifact. Note delayed recovery of sinus rhythm in the db/db mouse. **B**, Corrected SNRT (CSNRT) was obtained by subtracting the sinus cycle length from the SNRT and was significantly prolonged in db/db mice. **C**, Atrioventricular (AV) Wenckebach periodicity, measured as the shortest paced cycle length allowing conduction of electrical impulse from atria to ventricles, remained unchanged in db/db mice. **D**, Ventricular effective refractory period (VERP), measured by extra-stimuli technique as the longest paced cycle length that did not elicit a propagating response, was indistinguishable in db/+ and db/db mice. *, P < 0.05, Student’s *t*-test. Number of animals is indicated in parentheses. The SNRT and AV Wenckebach period were not obtained in 1 db/+ and 1 db/db mouse, respectively, due to technical difficulties.

To determine whether the origin of the cardiac arrhythmia was indeed the SAN, we evaluated the SAN directly in an invasive electrophysiological study. Sinus node recovery time was 139 ± 3.8 ms in db/+ mice, but delayed to 170 ± 13 ms in db/db mice (P < 0.05, Figure 5A). Sinus node recovery time corrected for baseline heart rate (CSNRT) also showed delayed recovery time in db/db mice (Figure 5B), indicating reduced SAN automaticity. Whereas db/db mice exhibited impaired SAN function, they show no change in AV Wenckebach periodicity (Figure 5C), a finding supported by preserved PQ intervals (Figure 2C), or VERP (Figure 5D). Susceptibility to pacing-induced ventricular arrhythmias were comparable in the two groups of mice (db/+: 3/11 versus db/db: 1/9 mice; P > 0.05).

### Reduced neuronal density in the sinoatrial node of db/db mice

**Figure 6:**
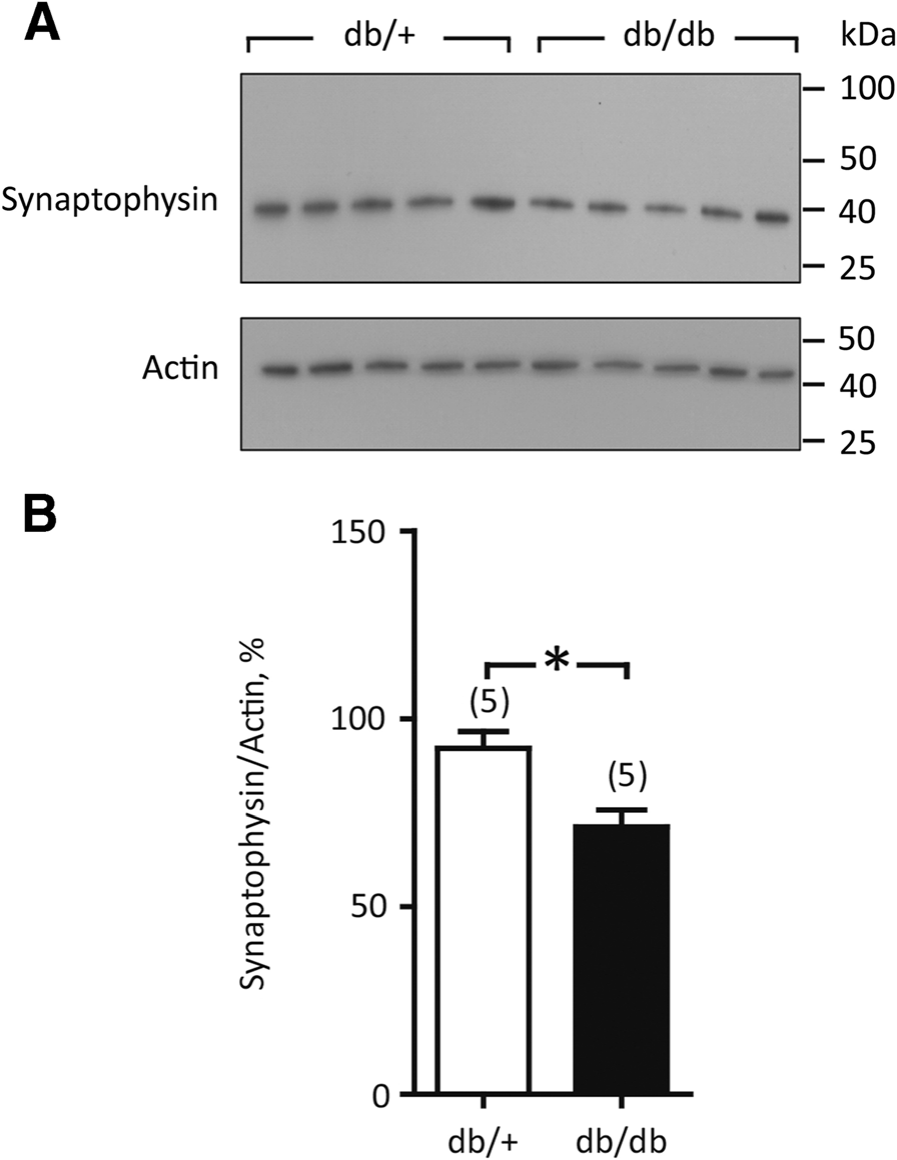
**Synaptophysin expression in right atria tissue. A**, Exemplary immunoblots. **B**, Averaged ratio of synaptophysin/actin band densities (5 hearts x 3 independent immunoblotting experiments). *, P < 0.05, Student’s *t*-test.

*In vivo*, pacemaking activity of the SA node is constantly modulated by an input from autonomic nervous system. Prolonged CSNRT in db/db mice would suggest intrinsic nodal abnormalities, but we suspected that SA nodal dysfunction may be extrinsic as it is not evident at the baseline ECG but is unmasked by adrenergic stimulation. Impaired autonomic modulation may be attributed to nerve rarefaction so we harvested right atrial tissue including SAN to quantify expression of synaptophysin. Synaptophysin migrated as a single band, at the expected molecular weight of approximately 40 kDa (Figure 6A). On average, db/db mice showed a 29% lower synaptophysin/actin ratio, suggesting diminished autonomic nerve density as a potential modulator of the intrinsic SAN dysfunction.

## Discussion

Increased heart rate is the common finding in patients with T2DM; however, diabetes is also associated with increased susceptibility to slow heart rates [7,12] and with abnormal chronotropic responses to stress [28], suggesting defective pacemaking function in some of these patients. Altered heart rate may contribute to excess cardiovascular mortality in T2DM [4,5] and it is important to unravel how SAN dysfunction is triggered and to find ways to prevent or treat it. In addition, clinical risk stratification of patients using heart rate and HRV may be misinterpreted in settings of SAN dysfunction and potentially in T2DM. Our data provides experimental evidence that T2DM complicated by obesity, adversely affects SAN function. In vivo, electrophysiological testing in db/db mice revealed substantial prolongation of corrected SAN recovery time, indicating abnormal pacemaking function. The defect in the SAN becomes manifest during fasting and pathologic during stress test with isoprenaline, where db/db mice exhibited SAN arrhythmia resulting in a profound fluctuation of RR intervals.

### Diabetic mice have SAN dysfunction

Luo and colleagues recently showed that mice with streptozotocin-induced T1DM also develop SAN dysfunction, which manifested as a reduced resting heart rate, prolonged CSNRT and an attenuated chronotropic response to isoprenaline in isolated, perfused hearts [15]. Rats with streptozotocin-induced T1DM also show signs of nodal abnormalities, such as bradycardia and prolongation of the SAN action potential [16,29,30]. In addition, an early experimental study of effects of diabetes on conduction system also demonstrated prolonged CSNRT in atrial preparations from alloxan-treated rabbits [17]. With the present study, we show that SAN dysfunction also is present in models of T2DM.

In the present study, the chronotropic response to isoprenaline was comparable in db/db and db/+ mice. Heart rate reached a maximal level 30–40 sec after the injection (Figure 3), but surprisingly, the arrhythmias in db/db mice were delayed till 5–10 minutes after adrenergic stimulation. Heart rate is determined by the intrinsic pacemaking activity of the SAN; however, it is modulated by external factors such as autonomic function, hemodynamic load and metabolic status [31,32]. The db/db mice show complex cardioregulatory autonomic dysfunction, hypertension and metabolic disturbances [18], which may contribute to or underlie the observed SAN arrhythmias. We found that the nodal defects in db/db mice coincide with a relative denervation of SAN as implicated by a 29% lower expression of synaptophysin in right atrial tissue (Figure 6), in accordance with earlier reports from an animal model of diabetic cardiac autonomic neuropathy [33]. In the present study, we have not proven a causal relationship between reduced nerve density in pacemaker tissue of db/db mice and abnormal heart rate fluctuations; however, since SAN arrhythmia was not present at the resting conditions, but developed as a consequence of a persistent autonomic challenge, it strongly suggest a contribution of defective autonomic SAN regulation and potentially neurotransmitter depletion in diabetes-related nodal dysfunction. This is supported by the clinical finding of cardiovascular autonomic neuropathy in up to 90% patients with advanced diabetes [34], and reports of compromised neurotransmitter handling in cardiac tissue from diabetic patients [35–37].

Isoprenaline is a mixed β-adrenergic receptor agonist that besides it’s positive chronotropic response via β_1_-adrenergic receptors in the SAN will elicit a β_2_-mediated relaxation of arterioles, resulting in a reduction of the peripheral blood pressure. Activation of the baroreceptors would augment the activity of the sympathetic nerves to the heart and reduce vagal activity. Spontaneous baroreflex sensitivity is comparable in db/db and db/+ mice [19], and it is not affected by the presence of diabetes in patients with coronary artery disease [38]. Notwithstanding, cardiac autonomic neuropathy and vagal remodeling is a serious complication of diabetes; however, animal studies have shown that cardiac cholinergic neurons and atrial sensitivity to acetylcholine are not lost [39]. In the present study, we have not monitored peripheral blood pressure and cannot comment on the potential impact of baroreceptor sensitivity or cardiac autonomic neuropathy on the observed RR fluctuations in db/db mice.

The mechanisms behind the observed SAN dysfunction remain elusive. Sinoatrial conduction time may be impaired due to lipid accumulation and lipotoxicity in hyperglycemic diabetes [40]. In addition, rats offered high-glucose drinking water for 8 weeks develop dysfunctional SAN and perinodal fat depositions, potentially explaining the elevated RR fluctuations in the animals [41]. Oxidative stress causing SAN cell death and fibrotic replacement via chronic activation of the calcium/calmodulin-dependent protein kinase II (CaMKII) has been suggested to contribute to increased sudden death in diabetic patients [15]. Moreover, hyperglycemia directly activates CaMKII in ventricular cardiomyocytes thereby aggravating calcium-dependent cardiac arrhythmias [42]. In the isoprenaline experiments of the present study, relative hyperglycemia and activation of the β-adrenergic signaling pathway may have conspired to activate CaMKII in the db/db SAN [43] thereby facilitating SAN arrhythmias. In the invasive studies, relative hypoglycemia and absent β-adrenoceptor stimulation may explain why overt RR fluctuations were not observed.

### Early hemodynamic remodeling in diabetic mice

Echocardiographic imaging of diastolic function in db/+ mice showed transmitral flow velocities comparable to earlier reports in wild-type mice [25]. Cardiac imaging in db/db mice showed diastolic insufficiency and increased systolic function. These alterations are similar to findings from patients with insulin resistance and early-stage diabetes that show diastolic dysfunction, but no signs of systolic impairment or structural changes [44,45]. The smaller weight of the db/db hearts is in part explained bý the shorter LV parasternal length. It is tempting to speculate that that the shorter LV length in db/db mice contributes to improved systolic function via a reduction of wall stress, according to La Place’s law. Several other studies have used cardiac imaging to evaluate cardiac structure and mechanical function in db/db model. It appears that no consensus is established as some reports show cardiac hypertrophy with systolic and diastolic dysfunction [46,47], whereas others reported no change in cardiac function [20]. Discrepancies between some reports may be due to the duration and the type of diabetes, background mouse strain, gender and anesthesia.

The present study was performed in leptin-receptor deficient mice characterized by high levels of circulating leptin. Leptin-deficient mice develop obesity and cardiac remodeling which is completely reversed by leptin infusion, suggesting that leptin itself has cardiac anti-hypertrophic bioactivity [22]. Notwithstanding, pro-hypertrophic effects of leptin has also been shown, primarily in vitro [48–50], but also in vivo [51]. It is not known whether high plasma levels of leptin, obesity or diabetes *per se* is the culprit for inducing the observed SAN dysfunction or the reduced autonomic nerve density.

### Electrocardiographic abnormalities in db/+ mice

We chose to use the genetically comparable, lean, non-diabetic db/+ mice as controls in the present study, rather than +/+ mice. The variability in the cardiac axis among the individual db/+ mice in the present study is surprisingly large (Figure 2A). The cardiac QRS axis is the general direction of the depolarization wave front in the ventricles, and would normally lie between 0 and 120° when the impulse spread from the AV node throughout the heart. Left and right sided axis deviation from the normal range are usually contributed to left or right ventricular hypertrophy, respectively, or a conduction defect. Excessive abdominal fat, as is often seen in T2DM patients, can displace the diaphragm and angle the heart towards an apparent left axis deviation, when the patient is in a supine position; however, this cannot be the underlying reason for the axis variability in the lean controls. It is beyond the scope of the present study to determine the reason for the highly inconsistent and abnormal cardiac axis among db/+ mice, but it raises a serious concern regarding whether the db/+ mouse is an appropriate control for the db/db mouse in studies of cardiac complications in the setting of diabetes.

### Clinical implications

The association between diabetes and defective pacemaker function in the heart may contribute to the increased cardiovascular mortality in the diabetic population. As diabetic patients are overrepresented in the population with electronic pacemakers treating intrinsically slow heart rates [7], experimental, translational and clinical studies of SAN function on a diabetic background are relevant. Diabetic patients may be at increased risk of life-threatening cardiac arrhythmias when exposed to periods of increased adrenergic drive in physical exercise or emotional stress. Future clinical studies should be designed to verify if diabetes constitutes a risk factor for development of SAN dysfunction and if so whether it’s early detection and treatment could lower mortality in diabetic patients.

### Limitations

The present study offers little insight into the time course of diabetes-related SAN dysfunction as we evaluated mice only at one time point. Synaptophysin is a general marker of nerve synapses, and we have not established a causal mechanism between synaptophysin downregulation and SAN dysfunction. Db/db mice are obese, hyperglycemic and diabetic and which metabolic alteration is the primarily driver for development of SAN dysfunction remains to be determined. Anesthesia reduces the power of HRV and conclusions from the frequency domain analysis should be drawn with caution [52]. Finally, limitations have to be considered when extrapolating experimental data from small animals to diabetic patients.

## Conclusions

Sinoatrial node dysfunction, characterized by prolonged CSNRT and autonomic nerve rarefaction, is present in mice with diabetes. β-Adrenoreceptor stimulation triggers cardiac arrhythmia originating in the SAN. Thus, dysfunction of the intrinsic cardiac pacemaker and remodeling of the autonomic nervous system may conspire to increase cardiac mortality in diabetic patients. Moreover, improved systolic function and reduced diastolic function indicates early ventricular remodeling secondary to obesity and T2DM in db/db mice.

## References

[1] Wild S, Roglic G, Green A, Sicree R, King H (2004). Global prevalence of diabetes estimates for the year 2000 and projections for 2030. Diabetes Care.

[2] Kannel WB, McGee DL (1979). Diabetes and glucose tolerance as risk factors for cardiovascular disease: the Framingham study. Diabetes Care.

[3] Donahoe SM, Stewart GC, McCabe CH, Mohanavelu S, Murphy SA, Cannon CP, Antman EM (2007). Diabetes and mortality following acute coronary syndromes. JAMA.

[4] Hillis GS, Woodward M, Rodgers A, Chow CK, Li Q, Zoungas S, Patel A, Webster R, Batty GD, Ninomiya T, Mancia G, Poulter NR, Chalmers J (2012). Resting heart rate and the risk of death and cardiovascular complications in patients with type 2 diabetes mellitus. Diabetologia.

[5] Linnemann B, Janka HU (2003). Prolonged QTc interval and elevated heart rate identify the type 2 diabetic patient at high risk for cardiovascular death: the Bremen diabetes study. Exp Clin Endocrinol Diabetes.

[6] Kataoka M, Ito C, Sasaki H, Yamane K, Kohno N (2004). Low heart rate variability is a risk factor for sudden cardiac death in type 2 diabetes. Diabetes Res Clin Pract.

[7] Hasslacher C, Wahl P (1977). Diabetes prevalence in patients with bradycardiac arrhythmias. Acta Diabetol Lat.

[8] Podlaha R, Falk A (1992). The prevalence of diabetes mellitus and other risk factors of atherosclerosis in bradycardia requiring pacemaker treatment. Horm Metab Res Suppl Ser.

[9] Grimm W, Langenfeld H, Maisch B, Kochsiek K (1990). Symptoms, cardiovascular risk profile and spontaneous ECG in paced patients: a five-year follow-up study. Pacing Clin Electrophysiol PACE.

[10] Wasada T, Katsumori K, Hasumi S, Kasanuki H, Arii H, Saeki A, Kuroki H, Saito S, Omori Y (1995). Association of sick sinus syndrome with hyperinsulinemia and insulin resistance in patients with non-insulin-dependent diabetes mellitus: report of four cases. Intern Med Tokyo Jpn.

[11] Mak KH, Moliterno DJ, Granger CB, Miller DP, White HD, Wilcox RG, Califf RM, Topol EJ (1997). Influence of diabetes mellitus on clinical outcome in the thrombolytic era of acute myocardial infarction: GUSTO-I investigators: global utilization of streptokinase and tissue plasminogen activator for occluded coronary arteries. J Am Coll Cardiol.

[12] Movahed M-R, Hashemzadeh M, Jamal MM (2005). Increased prevalence of third-degree atrioventricular block in patients with type II diabetes mellitus. Chest.

[13] Hummel KP, Dickie MM, Coleman DL (1966). Diabetes, a new mutation in the mouse. Science.

[14] Belke DD, Severson DL (2012). Diabetes in mice with monogenic obesity: the db/db mouse and its use in the study of cardiac consequences. Methods Mol Biol.

[15] Luo M, Guan X, Luczak ED, Lang D, Kutschke W, Gao Z, Yang J, Glynn P, Sossalla S, Swaminathan PD, Weiss RM, Yang B, Rokita AG, Maier LS, Efimov IR, Hund TJ, Anderson ME (2013). Diabetes increases mortality after myocardial infarction by oxidizing CaMKII. J Clin Invest.

[16] Howarth FC, Al-Sharhan R, Al-Hammadi A, Qureshi MA (2007). Effects of streptozotocin-induced diabetes on action potentials in the sinoatrial node compared with other regions of the rat heart. Mol Cell Biochem.

[17] Senges J, Brachmann J, Pelzer D, Hasslacher C, Weihe E, Kübler W (1980). Altered cardiac automaticity and conduction in experimental diabetes mellitus. J Mol Cell Cardiol.

[18] Goncalves AC, Da C, Tank J, Diedrich A, Hilzendeger A, Plehm R, Bader M, Luft FC, Jordan J, Gross V (2009). Diabetic hypertensive leptin receptor-deficient db/db mice develop cardioregulatory autonomic dysfunction. Hypertension.

[19] Senador D, Kanakamedala K, Irigoyen MC, Morris M, Elased KM (2009). Cardiovascular and autonomic phenotype of db/db diabetic mice. Exp Physiol.

[20] Daniels A, Van Bilsen M, Janssen BJA, Brouns AE, Cleutjens JPM, Roemen THM, Schaart G, van der Velden J, van der Vusse GJ, Van Nieuwenhoven FA (2010). Impaired cardiac functional reserve in type 2 diabetic db/db mice is associated with metabolic, but not structural, remodelling. Acta Physiol.

[21] Su W, Guo Z, Randall DC, Cassis L, Brown DR, Gong MC (2008). Hypertension and disrupted blood pressure circadian rhythm in type 2 diabetic db/db mice. Am J Physiol Heart Circ Physiol.

[22] Barouch LA, Berkowitz DE, Harrison RW, O’Donnell CP, Hare JM (2003). Disruption of leptin signaling contributes to cardiac hypertrophy independently of body weight in mice. Circulation.

[23] Yue P, Arai T, Terashima M, Sheikh AY, Cao F, Charo D, Hoyt G, Robbins RC, Ashley EA, Wu J, Yang PC, Tsao PS (2007). Magnetic resonance imaging of progressive cardiomyopathic changes in the db/db mouse. Am J Physiol Heart Circ Physiol.

[24] Speerschneider T, Grubb S, Metoska A, Olesen S-P, Calloe K, Thomsen MB (2013). Development of heart failure is independent of K + channel-interacting protein 2 expression. J Physiol.

[25] Schaefer A, Klein G, Brand B, Lippolt P, Drexler H, Meyer GP (2003). Evaluation of left ventricular diastolic function by pulsed Doppler tissue imaging in mice. J Am Soc Echocardiogr.

[26] Speerschneider T, Thomsen MB (2013). Physiology and analysis of the electrocardiographic T wave in mice. Acta Physiol.

[27] Thireau J, Zhang BL, Poisson D, Babuty D (2008). Heart rate variability in mice: a theoretical and practical guide. Exp Physiol.

[28] Abenavoli T, Rubler S, Fisher VJ, Axelrod HI, Zuckerman KP (1981). Exercise testing with myocardial scintigraphy in asymptomatic diabetic males. Circulation.

[29] Hicks KK, Seifen E, Stimers JR, Kennedy RH (1998). Effects of streptozotocin-induced diabetes on heart rate, blood pressure and cardiac autonomic nervous control. J Auton Nerv Syst.

[30] Zhang L, Parratt JR, Beastall GH, Pyne NJ, Furman BL (2002). Streptozotocin diabetes protects against arrhythmias in rat isolated hearts: role of hypothyroidism. Eur J Pharmacol.

[31] Camm AJ, Malik M, Bigger JT, Breithardt G, Cerutti S, Cohen RJ, Coumel P, Fallen EL, Kennedy HL, Kleiger RE, Lombardi F, Malliani A, Moss AJ, Rottman JN, Schmidt G, Schwartz PJ, Singer D (1996). Heart rate variability - standards of measurement, physiological interpretation, and clinical use. Circulation.

[32] Adán V, Crown LA (2003). Diagnosis and treatment of sick sinus syndrome. Am Fam Physician.

[33] Yang B, Chon KH (2011). Assessment of diabetic cardiac autonomic neuropathy in type I diabetic mice. Conf Proc Annu Int Conf IEEE.

[34] Vinik AI, Ziegler D (2007). Diabetic cardiovascular autonomic neuropathy. Circulation.

[35] Oberhauser V, Schwertfeger E, Rutz T, Beyersdorf F, Rump LC (2001). Acetylcholine release in human heart atrium: influence of muscarinic autoreceptors, diabetes, and age. Circulation.

[36] Neubauer B, Christensen NJ (1976). Norepinephrine, epinephrine, and dopamine contents of the cardiovascular system in long-term diabetics. Diabetes.

[37] Scognamiglio R, Casara D, Avogaro A (2000). Myocardial dysfunction and adrenergic innervation in patients with Type 1 diabetes mellitus. Diabetes Nutr Metab.

[38] Kiviniemi AM, Hautala AJ, Karjalainen JJ, Piira O-P, Lepojärvi S, Tiinanen S, Seppänen T, Ukkola O, Huikuri HV, Tulppo MP (2013). Impact of type 2 diabetes on cardiac autonomic responses to sympathetic stimuli in patients with coronary artery disease. Auton Neurosci Basic Clin.

[39] Mabe AM, Hoover DB (2011). Remodeling of cardiac cholinergic innervation and control of heart rate in mice with streptozotocin-induced diabetes. Auton Neurosci Basic Clin.

[40] Olsen KB, Axelsen LN, Braunstein TH, Sørensen CM, Andersen CB, Ploug T, Holstein-Rathlou N-H, Nielsen MS (2013). Myocardial impulse propagation is impaired in right ventricular tissue of Zucker diabetic fatty (ZDF) rats. Cardiovasc Diabetol.

[41] Albarado-Ibañez A, Avelino-Cruz JE, Velasco M, Torres-Jácome J, Hiriart M (2013). Metabolic syndrome remodels electrical activity of the sinoatrial node and produces arrhythmias in rats. PLoS ONE.

[42] Erickson JR, Pereira L, Wang L, Han G, Ferguson A, Dao K, Copeland RJ, Despa F, Hart GW, Ripplinger CM, Bers DM (2013). Diabetic hyperglycaemia activates CaMKII and arrhythmias by O-linked glycosylation. Nature.

[43] Wu Y, Gao Z, Chen B, Koval OM, Singh MV, Guan X, Hund TJ, Kutschke W, Sarma S, Grumbach IM, Wehrens XHT, Mohler PJ, Song L-S, Anderson ME (2009). Calmodulin kinase II is required for fight or flight sinoatrial node physiology. Proc Natl Acad Sci U S A.

[44] Schannwell CM, Schneppenheim M, Perings S, Plehn G, Strauer BE (2002). Left ventricular diastolic dysfunction as an early manifestation of diabetic cardiomyopathy. Cardiology.

[45] Rutter MK, Parise H, Benjamin EJ, Levy D, Larson MG, Meigs JB, Nesto RW, Wilson PWF, Vasan RS (2003). Impact of glucose intolerance and insulin resistance on cardiac structure and function: sex-related differences in the Framingham heart study. Circulation.

[46] Carley AN, Semeniuk LM, Shimoni Y, Aasum E, Larsen TS, Berger JP, Severson DL (2004). Treatment of type 2 diabetic db/db mice with a novel PPARgamma agonist improves cardiac metabolism but not contractile function. Am J Physiol Endocrinol Metab.

[47] Bartels ED, Nielsen JM, Bisgaard LS, Goetze JP, Nielsen LB (2010). Decreased expression of natriuretic peptides associated with lipid accumulation in cardiac ventricle of obese mice. Endocrinology.

[48] Rajapurohitam V, Gan XT, Kirshenbaum LA, Karmazyn M (2003). The obesity-associated peptide leptin induces hypertrophy in neonatal rat ventricular myocytes. Circ Res.

[49] Xu F-P, Chen M-S, Wang Y-Z, Yi Q, Lin S-B, Chen AF, Luo J-D (2004). Leptin induces hypertrophy via endothelin-1-reactive oxygen species pathway in cultured neonatal rat cardiomyocytes. Circulation.

[50] Madani S, De Girolamo S, Muñoz DM, Li R-K, Sweeney G (2006). Direct effects of leptin on size and extracellular matrix components of human pediatric ventricular myocytes. Cardiovasc Res.

[51] Abe Y, Ono K, Kawamura T, Wada H, Kita T, Shimatsu A, Hasegawa K (2007). Leptin induces elongation of cardiac myocytes and causes eccentric left ventricular dilatation with compensation. Am J Physiol Heart Circ Physiol.

[52] Kato M, Komatsu T, Kimura T, Sugiyama F, Nakashima K, Shimada Y (1992). Spectral analysis of heart rate variability during isoflurane anesthesia. Anesthesiology.

